# Coronary Artery Radial Deformation and Velocity in Native and Stented Arteries

**DOI:** 10.1155/2022/5981027

**Published:** 2022-03-26

**Authors:** Logan S. Schwarzman, Decebal S. Griza, Leon J. Frazin, Mladen I. Vidovich, Mayank M. Kansal

**Affiliations:** Division of Cardiology, University of Illinois at Chicago, Chicago, IL, USA

## Abstract

**Introduction:**

Coronary arteries are exposed to a variety of complex biomechanical forces during a normal cardiac cycle. These forces have the potential to contribute to coronary stent failure. Recent advances in stent design allow for the transmission of native pulsatile biomechanical forces in the stented vessel. However, there is a significant lack of evidence in a human model to measure vessel motion in native coronary arteries and stent conformability. Thus, we aimed to characterize and define coronary artery radial deformation and the effect of stent implantation on arterial deformation.

**Materials and Methods:**

Intravascular ultrasound (IVUS) pullback DICOM images were obtained from human coronary arteries using a coronary ultrasound catheter. Using two-dimensional speckle tracking, coronary artery radial deformation was defined as the inward and outward displacement (mm) and velocity (cm/s) of the arterial wall during the cardiac cycle. These deformation values were obtained in native and third-generation drug-eluting stented artery segments.

**Results:**

A total of 20 coronary artery segments were independently analyzed pre and poststent implantation for a total of 40 IVUS runs. Stent implantation impacted the degree of radial deformation and velocity. Mean radial deformation in native coronary arteries was 0.1230 mm ± 0.0522 mm compared to 0.0775 mm ± 0.0376 mm in stented vessels (*p*=0.0031). Mean radial velocity in native coronary arteries was 0.1194 cm/*s* ± 0.0535 cm/s compared to 0.0840 cm/*s* ± 0.0399 cm/s in stented vessels (*p*=0.0228).

**Conclusion:**

In this in vivo analysis of third-generation stents, stent implantation attenuates normal human coronary deformation during the cardiac cycle. The implications of these findings on stent failure and improved clinical outcomes require further investigation.

## 1. Introduction

In the human subject, coronary arteries are exposed to a number of various biomechanical forces during the normal cardiac cycle. Coronary twisting, stretching, compression, and rotation have all been described in various in vivo and in vitro studies [1–6]. It has been proposed that these biomechanical forces may have an effect on native coronary artery disease and the risk of stent failure in these patients [7]. Recent advances in stent manufacturing have targeted new devices that mitigate the potentially negative effects of stents on the coronary artery and allow for the preservation of native coronary motion. This is achieved by improved stent conformability via a mitigated impact of the stent on the mean radial deformation and mean radial velocity. Radial deformation is the compression and expansion of the coronary vessel lumen over a complete cardiac cycle, while radial velocity is the rate of change of this deformation per second. The objective benefit of these novel stents on the native biomechanical forces of coronary arteries of animal models, most commonly the porcine model, has been well established [5, 8–10]. Despite this research showing improvement for native vessel rotation in animal models, there remains a lack of a verified human model to measure radial deformation and velocity in vivo. The development of a human model to assess the effect of cardiac stents on coronary radial deformation and velocity has the potential to further understand and prevent coronary artery disease, improve stent conformability, and cultivate new coronary stent technology.

Thus, we aim to develop and verify a model for measuring coronary radial deformation and velocity in a human model and measure the effect of stent implantation on this biomechanical motion. This study assessed native and stented arteries using intravascular ultrasound imaging (IVUS) and a dedicated ultrasound imaging software to measure radial deformation and velocity in human coronary arteries.

## 2. Materials and Methods

### 2.1. Subject Enrollment

A total of 20 subjects were enrolled in the study. Subject enrollment was based on a retrospective analysis of patient electronic medical record data who were previously diagnosed with obstructive left anterior descending (LAD) coronary artery disease on cardiac catheterization and IVUS between January 1^st^, 2019 and June 1^st^, 2019. A total of 20 left anterior descending coronary artery segments were independently analyzed pre and poststent implantation for a total of 40 IVUS runs.

### 2.2. Definitions

The term coronary artery radial deformation was defined as the inward and outward deformation (mm) of a specified location of the lumen of the left anterior descending artery over a complete cardiac cycle. The term coronary artery radial velocity was defined as the inward and outward velocity (cm/s) of the arterial lumen during the cardiac cycle. Radial velocity was calculated as the rate of change of the radial deformation per second.

### 2.3. Experimental Design

#### 2.3.1. Intravascular Ultrasound Imaging (IVUS)

IVUS pullback images were obtained and analyzed using a coronary ultrasound catheter (Atlantis^®^ SR Pro 40 MHz Coronary Imaging Catheter; Boston Scientific, Natick, MA) and a commercially available measurement analytic system (iLab; Boston Scientific). Using fluoroscopy, the IVUS catheter was placed distal to the arterial portion of interest and an automated pullback was performed at a speed of 0.5 mm sec^−1^ covering the arterial portion mapped for stent implantation. The starting position of the IVUS catheter was determined by fluoroscopy using anatomical landmarks for reference points for IVUS pullback. Stationary IVUS imaging and analysis were completed at 4‐mm increments relative to the anatomical landmark for 3 full cardiac cycles. IVUS studies were archived onto DVDs for offline analysis.

#### 2.3.2. IVUS Assessment of Coronary Deformation and Velocity

IVUS images obtained from obstructive LAD coronary segments were converted into DICOM format and uploaded into a syngo® Ultrasound Workplace (Siemens Medical Solutions). The software measured the deformation of tissue motion by using 2D speckle tracking, which is an echocardiographic method based on the tracking of characteristic speckle patterns created by the constructive and destructive interference patterns of ultrasound beams in tissue [11]. The syngo® software performed speckle tracking via a technique termed vector velocity imaging whereby the motion was detected along a user‐defined tracing border by applying successive tracking steps [12]. From this tracking, radial deformation and velocity about the center of the region of interest can be determined. The software is typically used to evaluate myocardial strain and torsion from echocardiographic images. In this study, the software was utilized for a novel application to evaluate coronary artery radial deformation and velocity in a human coronary artery. Each subject was analyzed with the IVUS catheter in a stationary position for three full cardiac cycles in order to ensure consistency. Radial deformation and velocity measurements were obtained in obstructive coronary artery disease segments of native and third-generation drug-eluting stented left anterior descending coronary arteries.

### 2.4. Statistical Analysis

Statistical analysis was performed using SPSS (SPSS, Inc., version 26.0, Chicago, IL, USA). Mean and standard deviations were calculated for each of the runs. A two-tailed *T*-test was used to assess the statistical significance between the mean radial deformation and velocity in the two sample means. The two-tailed *T*-tests were calculated for the radial deformation and velocity in prestented compared to poststented coronary vessels. A T-score was determined from Student's t-distribution and represents the number of standard deviations the sample mean is from the population mean in a normal distribution. A T-score is particularly useful when evaluating a normal distribution in smaller sample sizes (*n* < 30) such as in this data set. A *p* value of <0.05 was considered as statistically significant.

## 3. Results

**Figure 1:**
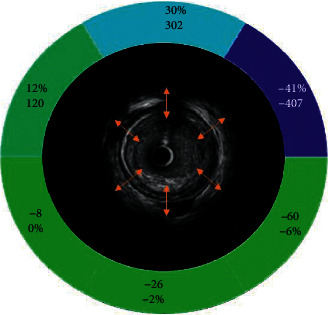
Representative image of the IVUS prestent implantation cross section. Representative image of the IVUS prestent implantation cross section that was measured using strain speckle tracking software. The left anterior descending vessel wall was divided into six 60-degree colored segments. The average of the six segments was calculated for total coronary vessel motion. The orange lines represent inward and outward motion (radial deformation) and radial velocity that was measured for each of the six vessel wall segments.

**Figure 2:**
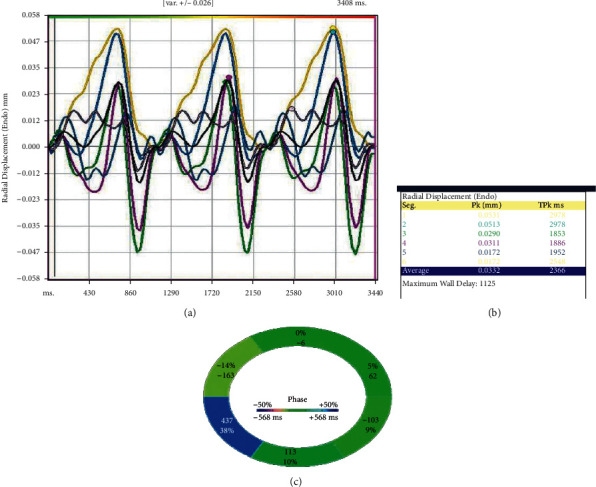
Poststent coronary artery vessel wall radial deformation. Example of syngo® software output for radial deformation (displacement) values over three full cardiac cycles. The colored curves represent regional deformation in a six-segment model of the coronary vessel. The black curve represents the averaged deformation of all of the individual segments.

**Figure 3:**
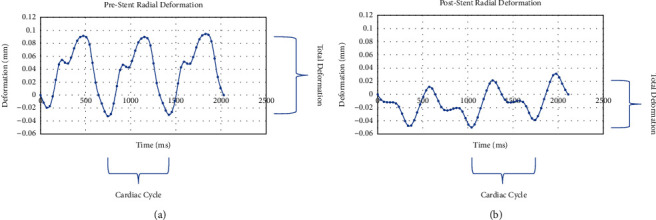
Pre and poststent coronary artery vessel wall radial deformation: (a) Prestent and (b) Poststent. Representative example of radial deformation over time in (a) prestent and after (b) poststent implantation. Poststent delivery, total coronary vessel radial deformation is attenuated compared to prestent delivery.

A total of 20 coronary artery segments were analyzed pre and poststent implantation for a total of 40 IVUS runs.  Figure 1 represents an image of the IVUS prestent implantation cross section that was measured using strain speckle tracking software of the six individual colored vessel wall segments.  Figure 2 represents an example of the syngo® software output for radial deformation values over three full cardiac cycles. Six individual colored segments of the coronary vessel deformation are graphed with the black curve representing the average deformation of all six segments. A representative example of prestent (A) and poststent (B) coronary artery vessel wall radial deformation is displayed in Figure 3. Systole occurs when the slope of the radial deformation curve is positive from trough to peak. Diastole occurs when the slope of the radial deformation curve is negative from peak to trough. Total coronary vessel radial deformation is attenuated in poststent compared to prestented coronary vessels.

Table 1 contains coronary artery wall radial deformation (mm) and velocity (cm/s) comparing pre and poststented vessels. Mean radial deformation in prestent vessels was 0.123 mm ± 0.052 mm compared to poststented vessels at 0.078 mm ± 0.038 mm (*p*=0.003). Mean coronary artery wall total velocity (cm/s) in prestent vessels was 0.119 cm/*s* ± 0.054 cm/s compared to poststented vessels at 0.084 cm/*s* ± 0.040 cm/s (*p*=0.023). A two-tailed *T*-test was used to calculate the velocity prestented compared to a poststented vessel. Inward velocity pre and poststent displayed a T-score of 2.73 (*p*=0.010) while outward velocity pre and poststent displayed a T-score of 1.923 (*p*=0.062).

## 4. Discussion

This study describes the impact of stent implantation on the coronary artery vessel wall motion. Our main findings are as follows: (1) assessing the effects of cardiac stents on coronary radial deformation and velocity in a human model is feasible, and (2) there is a significant reduction in coronary artery vessel wall motion deformation and velocity in poststented vessels compared to prestented vessels.

The use of IVUS to measure coronary artery rotation was first described by Challa et al. using a porcine model [6]. This study demonstrated that IVUS technology had the ability to accurately measure multiple data points on the porcine coronary vessel and track the motion of these data points over the course of a full cardiac cycle. In our study, we used a similar approach in human coronary arteries in order to measure the coronary radial deformation and velocity. This novel approach for human coronaries allows for an objective measurement of coronary vessel motion prior to and after stent implantation. It has been well described that native coronary vessels undergo a number of biomechanical forces, increasing the risk for metal stent fractures or restenosis [13–15]. Coronary stent technology strives towards greater stent conformability through novel engineering design features that promote native coronary vessel wall motion poststent implantation [16, 17]. This manuscript provides a method for objective measurement of the native coronary vessel wall motion pre and poststent via coronary artery vessel wall motion deformation and velocity measurement, which may be utilized by novel stent developers.

For the first time in a human model, we have demonstrated that current drug eluting stent technology significantly reduces coronary artery radial deformation and velocity compared to native coronary vessels. These findings are consistent with the porcine model findings previously described [6]. While additional studies with a greater sample size are necessary to discern the true cause, it can be hypothesized that drug eluting stent technology utilizes a metal strut frame which inhibits a number of the native coronary vessel biomechanical forces. Previous studies have described a complex array of coronary vessel twisting, stretching, compression, and rotation during the normal cardiac cycle which may increase the risk of stent restenosis or fracture [1–6, 18–20]. These forces are likely inhibited by stent technology due to the inherent properties of the metal. Our data supports these findings with a statistically significant reduction of both inward and outward deformation in precompared to poststented vessels (*p*=0.003 and *p*=0.023, respectively). Similarly, we observed a significant reduction of inward velocity in poststented compared to prestented vessels (*p*=0.010). Similarly, while the data showed a reduction in the outward velocity in poststent compared to prestent vessels, there was insufficient evidence to show statistical significance (*p*=0.062). It is possible that this difference in inward compared to outward velocity is due to coronary vessel filling which is attenuated by the metal stent structure. However, additional imaging to correct for these variations would be needed to confirm our hypothesis. Similar hypotheses were described for newer bioabsorbable scaffold technology with the complex biomechanical forces impacting the scaffold architecture leading to possible restenosis or stent fracture [21, 22]. Thus, this human model study using IVUS technology to measure coronary vessel radial deformation and velocity may have significant importance for future coronary stent design and measurement of stent conformability on the pre and poststented vessel.

To our knowledge, this study is the first human model to assess the impact of cardiac stents on coronary wall radial deformation and velocity using a previously porcine model-verified approach. Despite these strengths, there remain several limitations to our study. First, due to the small sample size, there needs to be further investigation to confirm these results. Secondly, our study utilized a retrospective analysis of human coronary vessels which may have introduced additional variability within the data. Third, the IVUS catheter may be off axis during pullback imaging within the coronary vessel. As a result, it is unclear whether the IVUS imaging probe was centered during the entirety of the run and may have introduced errors in our results. Fourth, there is a known limitation of through plane motion in strain imaging. The speckle tracking software is specifically designed to understand the ‘loss' and ‘gain' of ultrasonic speckles from frame to frame that occur due to this motion. Thus, small changes in the millimeter range are expected for any moving cardiac structure being imaged. The software takes this into account by tracking a ‘finger-print' of speckles from frame to frame, thus allowing for accurate tracking of vessel motion despite concurrent out-of-plane or through-plane motion. Fifth, images in this study were obtained using the Atlantis® SR Pro 40 MHz Coronary Imaging Catheter. This has recently been replaced with a 60 MHz model which produces higher resolution images and thus may impact the reported changes in diameter obtained in our study. Additional studies comparing the newer 60 MHz model with the 40 MHz model could be useful to assess the impact of image quality and device resolution on the clinical impact of various stent properties and help guide future IVUS and OCT studies. Of note, a recent study employing speckle-tracking on IVUS images demonstrated the favorable coronary rotational changes of an ‘uncaging' drug-eluting bioadaptor stent [23]. Lastly, as the IVUS images are not gated to the cardiac cycle, there may be additional variability in the determination of maximum systolic and diastolic deformation values. Peaks and troughs of the data were used to best estimate the radial deformation values during systole and diastole. Despite this, the consistent associations seen pre and poststent implantation suggest that this may not be a significant limitation.

## 5. Conclusions

In summary, we have developed an in vivo model for measuring coronary radial deformation and velocity. This allows for measuring the effects of stent implantation on native coronary artery biomechanical motion, which allows for a greater understanding of the mechanical forces on the coronary vessel. Current third-generation stent technology attenuates these complex biomechanical forces put upon a coronary artery during the normal cardiac cycle. The implications of these findings on stent failure and improved clinical outcomes require further investigation.

## Figures and Tables

**Table 1:** Coronary artery wall radial deformation (mm) and velocity (cm/s).

		Prestent	Poststent
Total deformation (mm)			
	**Mean**	0.123	0.078
	**Standard deviation**	0.522	0.038
		**Inward**	**Outward**
	***T*-Score**	3.163	2.374
	*p * **value**	0.003	0.023
**Total velocity (cm/s)**			
	**Mean**	0.119	0.084
	**Standard deviation**	0.054	0.040
		**Inward**	**Outward**
	***T*-Score**	2.731	1.923
	*p * **value**	0.010	0.062

## Data Availability

The data are available from the corresponding author on reasonable request.
